# Higher risk of SARS-CoV-2 Omicron BA.4/5 infection than of BA.2 infection after previous BA.1 infection, the Netherlands, 2 May to 24 July 2022

**DOI:** 10.2807/1560-7917.ES.2023.28.7.2200724

**Published:** 2023-02-16

**Authors:** Stijn P Andeweg, Brechje de Gier, Harry Vennema, Ivo van Walle, Noortje van Maarseveen, Nina E Kusters, Hester E de Melker, Susan JM Hahné, Susan van den Hof, Dirk Eggink, Mirjam J Knol

**Affiliations:** 1 Center for Infectious Disease Control, WHO COVID-19 reference laboratory, National Institute for Public Health and the Environment (RIVM), Bilthoven, The Netherlands; 2 Department of Medical Microbiology, University Medical Center Utrecht, Utrecht, the Netherlands; 3 Saltro Diagnostic Center for Primary Care, Utrecht, The Netherlands

**Keywords:** SARS-CoV-2, Omicron BA.2, Omicron BA.4, Omicron BA.5, Vaccination, previous infection

## Abstract

**Background:**

In summer 2022, SARS-CoV-2 Omicron BA.5 became dominant in Europe. In vitro studies have shown a large reduction of antibody neutralisation for this variant.

**Aim:**

We aimed to investigate differences in protection from previous infection and/or vaccination against infection with Omicron BA.4/5 vs BA.2.

**Methods:**

We employed a case-only approach including positive PCR tests from community testing between 2 May and 24 July 2022 that were tested for S gene target failure (SGTF), which distinguishes BA.4/5 from BA.2 infection. Previous infections were categorised by variant using whole genome sequencing or SGTF. We estimated by logistic regression the association of SGTF with vaccination and/or previous infection, and of SGTF of the current infection with the variant of the previous infection, adjusting for testing week, age group and sex.

**Results:**

The percentage of registered previous SARS-CoV-2 infections was higher among 19,836 persons infected with Omicron BA.4/5 than among 7,052 persons infected with BA.2 (31.3% vs 20.0%). Adjusting for testing week, age group and sex, the adjusted odds ratio (aOR) was 1.4 (95% CI: 1.3–1.5). The distribution of vaccination status did not differ for BA.4/5 vs BA.2 infections (aOR = 1.1 for primary and booster vaccination). Among persons with a previous infection, those currently infected with BA4/5 had a shorter interval between infections, and the previous infection was more often caused by BA.1, compared with those currently infected with BA.2 (aOR = 1.9; 95% CI: 1.5–2.6).

**Conclusion:**

Our results suggest immunity induced by BA.1 is less effective against BA.4/5 infection than against BA.2 infection.

**Key public health message**

**What did you want to address in this study?**

We investigated whether previous infection and/or vaccination against SARS-CoV-2 provides different protection against a new infection with the Omicron BA.4/5 or BA.2 variant.

**What have we learnt from this study?**

In our sample, previous SARS-CoV-2 infection protected better against infection with the BA.2 than the BA.4/5 variant. This indicates that BA.4/5 is better at evading infection-induced immunity. In contrast, vaccination protected equally well against infection with either variant.

**What are the implications of your findings for public health?**

Different immune evasion by variants within the Omicron lineage allows for repeated SARS-CoV-2 Omicron infections to occur. Such studies about immune evasion of current and novel virus variants are informative for developing updated vaccines.

## Background

The severe acute respiratory syndrome coronavirus 2 (SARS-CoV-2) Omicron (Phylogenetic Assignment of Named Global Outbreak (Pango) lineage designation: B.1.1.529) variants have caused large numbers of infections globally [1], driven by increased transmissibility and escape from vaccine- and infection-induced immunity [2-4]. Sub-variants of Omicron, mainly BA.1, BA.2, and BA.5, have been circulating globally. In the Netherlands, as in the rest of Europe, an initial BA.1 surge started at the end of 2021, rapidly succeeded by BA.2 in early 2022 and BA.4 and BA.5 from May 2022 [5]. Omicron variants have a large number of substitutions in the spike protein compared with earlier variants of concern (VOC), and the spike proteins of different Omicron lineages differ substantially from each other [6]. All Omicron variants show reduced sensitivity to SARS-CoV-2 antibodies induced by vaccination, previous infection or both (hybrid immunity). The spike substitutions in BA.4/5 cause the largest reduction in neutralisation [7-9], raising concern about the protection by vaccination and/or previous infection, including protection conferred by previous infections with other Omicron lineages.

To examine a possible reduction in protection from vaccine- and/or infection-induced immunity against BA.4/5 infection compared with protection against BA.2 infection, we employed a case-only approach to study the effect of pre-infection immune status (based on previous infection and/or vaccination) on the occurrence of BA.4/5 vs BA.2 infection during the transition period from BA.2 to BA.4/5 circulation (2 May to 24 July 2022). In addition, we assessed whether the interval between previous and current SARS-CoV-2 infection differed by variant, and we investigated the effect of different variants in the previous infection on the occurrence of BA.4/5 vs BA.2 infection.

## Methods

### Epidemiological data

From 1 June 2020 onwards, mass community testing for SARS-CoV-2, organised by the 25 regional public health services, has been available and advised for Dutch citizens experiencing coronavirus disease (COVID-19)-like symptoms. Since 11 April 2022, mostly individuals at high risk of severe disease and healthcare workers have been advised to still visit the public health service for PCR testing [10]. To assess S gene target failure (SGTF, see below), we used tests that were positive in national SARS-CoV-2 community testing with the TaqPath COVID-19 RT-PCR kit (ThermoFisher Scientific, Nieuwegein, the Netherlands) from 2 May to 24 July 2022. If a person had multiple tests positive for SGTF within 30 days during the study period, we included only the first positive test. Otherwise, both tests were included (this was only the case for one individual in our study).

Test results were linked to the national community testing register (CoronIT) using a unique sample number. This register contains pseudonymised data with demographic characteristics and self-reported vaccination status. We used these data to classify cases in different categories according to their vaccination status and whether they had a previous infection at least 30 days before the current infection, as previously described [3].

### BA.2 and BA.4/5 variant detection using S gene target failure

The TaqPath COVID-19 RT-PCR tests for three targets (S, ORF1ab and N) and is used by several laboratories in the Netherlands for SARS-CoV-2 diagnostics. In combination with a proper signal (quantification cycle ≤ 30) from ORF1ab and N targets, SGTF – also referred to as ‘S-gene not detected’ or S-dropout – has proven to be a highly specific proxy for SARS-CoV-2 variants containing the 69/70 deletion in the S gene. The SARS-CoV-2 variants Alpha (B.1.1.7) [11,12], Omicron BA.1 [4], BA.4 and BA.5 variants possess the S 69/70 deletion [13], while the ancestral strains, Beta (B.1.351), Gamma (P.1), Delta (B.1.617.2) [4] and Omicron BA.2 [3] do not possess the deletion [13]. The TaqPath PCR cannot distinguish BA.4 from BA.5, therefore we refer to SGTF as BA.4/5 and to non-SGTF as BA.2. The SGTF can only be used as a specific proxy for the variant when different variants containing or lacking the 69/70 deletion are not co-circulating or are only observed together for a very short period of time (see below). Whole genome sequencing (WGS) of a random selection of SGTF samples included in this study confirmed 52 (13.5%) BA.4 and 322 (83.6%) BA.5 cases among 385 sequenced SGTF samples, adding up to a positive predictive value of 97.1% (374/385) of SGTF to detect BA.4/5. The proportion of BA.5 among SGTF viruses increased over time. Non-SGTF results were strongly associated with BA.2 (including BA.2 sub-variants; positive predictive value of 98.0% (485/495) during the study period).

### Variant detection of previous infections

The variant causing the previous infection was determined if a WGS or SGTF result was available (1,609/7,625 previous infections; 21.1%). We used WGS to determine the variant in 117 of 1,609 (7.3%) previous infections with variant data. For the other 1,492 of 1,609 (92.7%), the variant was defined by SGTF result in combination with the testing date. Time periods were defined for SGTF and non-SGTF in which one variant could be called with ≥ 90% accuracy as co-circulation of different variants with the same SGTF/non-SGTF signal were minimal. This was done by grouping WGS-typed samples by their expected SGTF or non-SGTF status. The following periods were defined for the pre-VOC (18 January (start data collection) to 17 February 2021, non-SGTF), Alpha (18 January to 27 September 2021, SGTF), Delta (20 June 2021 to 7 January 2022, non-SGTF), Omicron BA.1 (23 November 2021 to 9 April 2022, SGTF) and Omicron BA.2 (29 January 2022 to end study, non-SGTF) variants. We provide an additional figure to display the periods where SGTF and non-SGTF results were used to detect the variant of previous infection (Supplementary Figure S1). No periods were defined for the Beta and Gamma variants as they were indistinguishable from pre-VOC, Delta and each other using SGTF with the ≥ 90% threshold.

### Statistical analysis

We included the following immune status groups: unvaccinated cases with and without a registered previous infection, cases with completed primary vaccination course and with and without a registered previous infection, and booster-vaccinated cases with and without a registered previous infection. Among persons with known immune status, we performed logistic regression to estimate the association between immune status, with unvaccinated without a previous infection as reference, and BA.4/5 vs BA.2 infection, adjusting for testing week, age group (18–29, 30–49, 50–69 and ≥ 70 years) and sex. If the Omicron BA.2 and BA.4/5 variants had similar ability to escape immunity from vaccination and/or previous infection, we would expect them to occur equally frequently in vaccinated and/or previously infected persons as in individuals without vaccination and previous infection, i.e. an odds ratio (OR) of 1. An OR > 1 would mean that BA.4/5 variants have better ability to escape immunity from vaccination and/or previous infection than the BA.2 variant. By adjusting for testing week, we compared BA.2 and BA.4/5 infections at the same moment in calendar time, thereby making a fair comparison between BA.2 and BA.4/5 cases in terms of effects of time since previous infection or vaccination.

Subsequently, we performed logistic regression to estimate the association between previous infection (ignoring vaccination status), with cases without a registered previous infection as reference, and BA.4/5 vs BA.2 infection, adjusting for testing week, age group (18–29, 30–49, 50–69 and ≥ 70 years) and sex. Among cases with a previous infection, we calculated the interval between testing dates in days and tested the difference in intervals between BA.2 and BA.4/5 infection with a Mann–Whitney test. The difference in the distribution of variants causing the previous infection between cases with current BA.2 and BA.4/5 infection was tested with a chi-squared test. Lastly, we performed logistic regression to estimate the association between variant of previous infection, with cases without a registered previous infection as reference, and BA.4/5 vs BA.2 infection, adjusting for testing week, age group (18–29, 30–49, 50–69 and ≥ 70 years) and sex.

## Results

**Figure 1:**
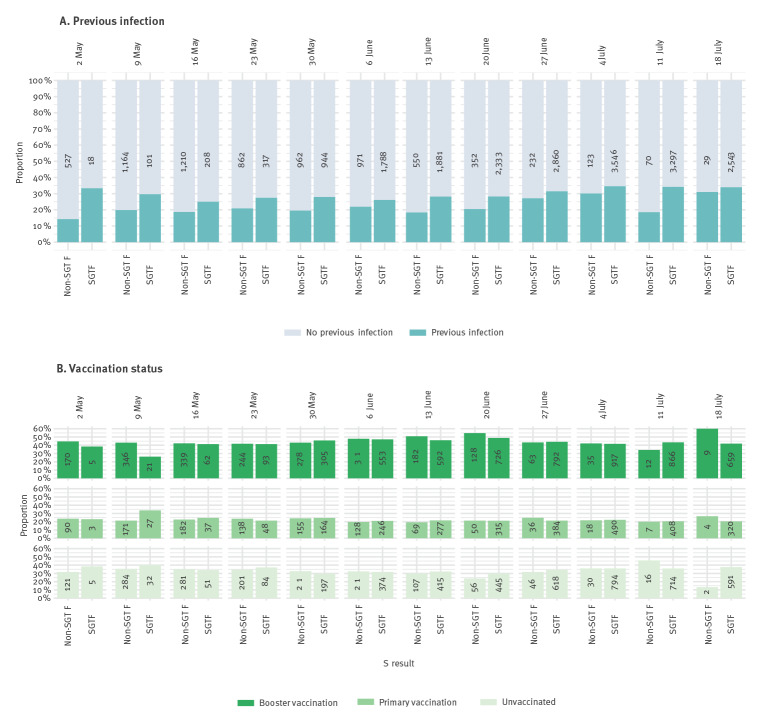
Proportion of cases infected with SARS-CoV-2 BA.2 (non-SGTF) and BA.4/5 (SGTF) who had previous infections (n = 26,888) and different vaccination histories (n = 17,391), per week, the Netherlands, 2 May–24 July 2022

SARS-CoV-2: severe acute respiratory syndrome coronavirus 2; SGTF: S gene target failure.

Between 2 May and 24 July 2022, 7,052 (26.2%) BA.2 and 19,836 (73.8%) BA.4/5 cases were detected (Table 1). In the first week, BA.4/5 comprised 18 of 545 (3.3%) of cases and in the last week 2,543 of 2,572 (98.9%). We provide an additional graphical representation of the BA.2 to BA.4/5 transition in Supplementary Figure S2. BA.4/5 cases were generally younger than BA.2 cases (Table 1). The proportion of cases with a previous infection was larger among BA.4/5 cases overall (Table 1) and per week during this period (Figure 1A). Of the 19,836 BA.4/5 cases, 6,215 (31.3%) had a previous infection compared with 1,408 of 7,052 (20.0%) BA.2 cases. No such differences were observed for vaccination status overall (Table 1) and per week (Figure 1B).

**Table 1:** Characteristics of cases with SARS-CoV-2 infection, by SGTF status, the Netherlands, 2 May–24 July 2022 (n = 26,888)

Level	S gene detected (BA.2)		S gene target failure (BA.4/5)	
	n	%	n	%
Total	7,052		19,836	
Age group (years)				
0–17	341	4.8	1,136	5.7
18–29	1,099	15.6	3,490	17.6
30–49	2,542	36.0	7,099	35.8
50–69	2,469	35.0	6,385	32.2
≥ 70	596	8.5	1,703	8.6
Unknown	5	0.1	23	0.1
Sex				
Male	3,015	42.8	8,487	42.8
Female	4,026	57.1	11,326	57.1
Unknown	11	0.2	23	0.1
Week of testing in 2022				
2–8 May	527	7.5	18	0.1
9–15 May	1,164	16.5	101	0.5
16–22 May	1,210	17.2	208	1.0
23–29 May	862	12.2	317	1.6
30 May–5 June	962	13.6	944	4.8
6–12 June	971	13.8	1,788	9.0
13–19 June	550	7.8	1,881	9.5
20–26 June	352	5.0	2,333	11.8
27 June–3 July	232	3.3	2,860	14.4
4–10 July	123	1.7	3,546	17.9
11–17 July	70	1.0	3,297	16.6
18–24 July	29	0.4	2,543	12.8
Previous infection status				
No previous infection	5,644	80.0	13,621	68.7
Previous infection	1,408	20.0	6,215	31.3
Vaccination status				
Other (> 3 doses)	318	4.5	1,053	5.3
Booster vaccination	2,117	30.0	5,591	28.2
Primary vaccination	1,048	14.9	2,719	13.7
Partial vaccination	110	1.6	355	1.8
Unvaccinated	1,566	22.2	4,320	21.8
Unknown	1,893	26.8	5,798	29.2
Previous infection and vaccination status				
Previous infection, other vaccination (> 3 doses)	12	0.2	45	0.2
No previous infection, other vaccination (> 3 doses)	306	4.3	1,008	5.1
Previous infection, booster	178	2.5	918	4.6
No previous infection, booster	1,939	27.5	4,673	23.6
Previous infection, primary vaccination	253	3.6	1,146	5.8
No previous infection, primary vaccination	795	11.3	1,573	7.9
Previous infection, unvaccinated	609	8.6	2,394	12.1
No previous infection, unvaccinated	957	13.6	1,926	9.7
Unknown	2,003	28.4	6,153	31.0

SARS-CoV-2: severe acute respiratory syndrome coronavirus 2; SGTF: S gene target failure.

The number of cases corresponding to each group is displayed within the bars.

The higher proportion of previous infections among BA.4/5 cases remained after adjustment for testing week, age and sex, and the effect was present in unvaccinated and vaccinated cases (aOR ranging from 1.4 (95% CI: 1.2–1.7) in unvaccinated cases to 1.6 (95% CI: 1.3–2.0) in cases with primary or booster vaccination; Table 2). Among cases without a previous infection, there was no association between vaccination status and BA.4/5 vs BA.2 infection (aOR for primary vaccination: 1.1 (95% CI: 0.9–1.3); aOR for booster vaccination: 1.1 (95% CI: 1.0–1.3); Table 2).

**Table 2:** Association between immune status and SARS-CoV-2 BA.4/5 vs BA.2 infection in individuals ≥ 18 years, adjusted for testing week, sex and age group^a^, the Netherlands, 2 May–24 July 2022 (n = 16,178)

	S gene detected (BA.2)		S gene target failure (BA.4/5)		Adjusted OR (95% CI)
	n	%	n	%	
No previous infection, unvaccinated	853	19.2	1,646	14.0	Reference
Previous infection, unvaccinated	507	11.4	1,973	16.8	1.4 (1.2–1.7)
No previous infection, primary vaccination	736	16.5	1,441	12.3	1.1 (0.9–1.3)
Previous infection, primary vaccination	241	5.4	1,095	9.3	1.6 (1.3–2.0)
No previous infection, booster	1,937	43.5	4,658	39.7	1.1 (1.0–1.3)
Previous infection, booster	177	4.0	914	7.8	1.6 (1.3–2.0)

CI: confidence interval; OR: odds ratio; SARS-CoV-2: severe acute respiratory syndrome coronavirus 2.

^a^ 18–29, 30–49, 50–69 and ≥ 70 years.

**Figure 2:**
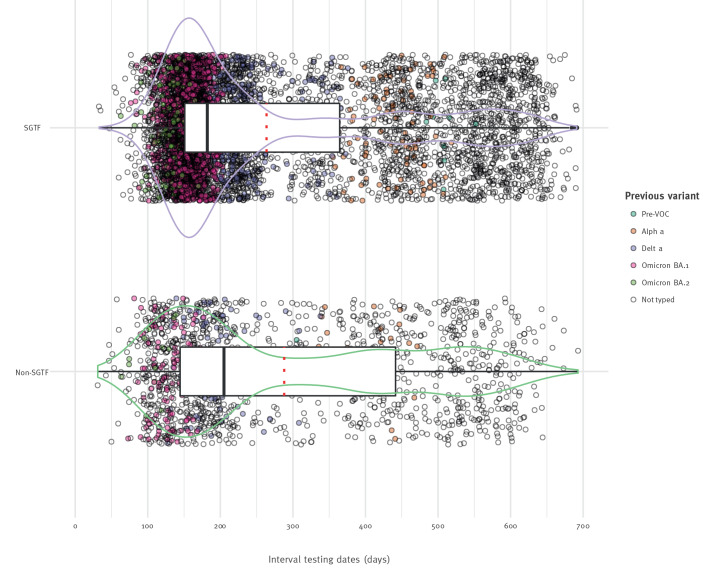
Interval of testing dates between previous and current infections with SARS-CoV-2 BA.2 (non-SGTF) and BA.4/5 (SGTF), in days, the Netherlands, 2 May–24 July 2022 (n = 7,625)

SGTF: S gene target failure; SARS-CoV-2: severe acute respiratory syndrome coronavirus 2; VOC: variant of concern.

Overall, previous infection was associated with an increased risk of infection with BA.4/5 compared with BA.2, after adjustment for testing week, age and sex with an aOR of 1.4 (95% CI: 1.3–1.5; Table 3). Among cases with previous infections, intervals between infections were shorter in BA.4/5 cases (median interval: 182 days) compared with BA.2 cases (median interval 206 days, p value 0.004, Figure 2). Previous BA.1 infection was more frequent in BA.4/5 cases than in BA.2 cases (62.5% and 44.3%, respectively, p < 0.001, Table 3). We performed an additional stratification by testing week of the current infection, in which the proportion of previous BA.1 infections was higher among BA.4/5 cases than BA.2 cases and this analysis is made available in Supplementary Figure S3, indicating that BA.4/5 has a higher capacity to escape protection conferred by BA.1 infection compared with other previous infections. Indeed, when adjusting for testing week, age and sex, previous BA.1 infection in particular was associated with BA.4/5 rather than BA.2 infection (aOR: 1.9; 95% CI: 1.5–2.6). For previous infection with all other variants, except BA.2, the risk of BA.4/5 was greater than of BA.2 infection (Table 3). A complete case analysis, removing the cases without known vaccination status from the previous infection analyses, resulted in similar outcomes: the intervals between infections were shorter for BA.4/5 than BA.2 (p value < 0.001; details are appended in Supplementary Figure S4), previous infection was associated with an increased risk of BA.4/5 infection compared with the risk for infection with BA.2, and the percentage of previous BA.1 infection among BA.4/5 current infections was higher than among BA.2 current infections (63.2% vs 49.7%, p value = 0.002; see Supplementary Table S1 for the detailed data).

**Table 3:** Association between previous SARS-CoV-2 infection (n = 25,377, top), or previous variant (n = 19,896, bottom), and BA.4/5 vs BA.2 infection, the Netherlands, 2 May–24 July 2022

	S gene detected (BA.2)		S gene target failure (BA.4/5)		Adjusted OR (95% CI)
	n	%	n	%	
By previous infection					
No previous infection	5,423	80.9	13,049	69.9	Reference
Previous infection	1,277	19.1	5,628	30.1	1.4 (1.3–1.5)
By variant of previous infection					
No previous infection	5,423	96.6	13,049	91.4	Reference
Pre-VOC	1	0	8	0.1	2.3 (0.2–70.2)
Alpha	25	0.4	80	0.6	1.6 (0.9–2.9)
Delta	69	1.2	289	2	1.3 (1.0–1.9)
Omicron BA.1	85	1.5	770	5.4	1.9 (1.4–2.6)
Omicron BA.2	12	0.2	85	0.6	0.6 (0.3–1.4)

CI: confidence interval; OR: odds ratio; SARS-CoV-2: severe acute respiratory syndrome coronavirus 2; VOC: variant of concern.

Analyses are in individuals aged 18 years and older, adjusted for testing week, sex and age group (18–29, 30–49, 50–69 and ≥ 70 years).

Current infections are coloured to indicate the variant of the previous infection. Median is displayed in black (line) and mean in red (dotted). Data density is represented with a violin plot in purple for SGTF (BA.4/5) and in green for non-SGTF (BA.2).

## Discussion

Evidence of escape of infection- and/or vaccination-induced immunity by novel SARS-CoV-2 VOC is highly relevant for vaccine policy. We found that Omicron BA.4/5 cases more often had previous infections than BA.2 cases when adjusted for week of testing, irrespective of vaccination status. This indicates relatively more evasion of infection-induced immunity by the BA.4/5 than BA.2 variant. There was no association between vaccination status and BA.4/5 infection vs BA.2 infection, suggesting that vaccination induced equal protection against the BA.4/5 and BA.2 variants. Among cases with a previous infection, we found shorter intervals between previous and current infection for BA.4/5 than for BA.2 infections. In addition, in individuals with a previous BA.1 infection, the risk of a BA.4/5 reinfection was significantly higher than of BA.2 reinfection. This suggests that BA.1-induced immunity protected less well and/or shorter against BA.4/5 infection than against BA.2 infection.

In vitro studies have shown that the BA.4/5 variant can escape neutralising antibodies elicited by vaccination and by infection with the Omicron variant (BA.1 or BA.2) [7-9]. A study from Denmark showed no difference in vaccine effectiveness between BA.5 and BA.2 [14], which is in line with our results. That study observed a protective effect of previous Omicron BA.1/2 infection of 92.7% (95% CI: 91.6–93.7) and 97.1% (95% CI: 96.6–97.5) against BA.5 and BA.2 infection, respectively. The difference between these effects, a ratio of 2.5, corresponds broadly with our results, as we found an OR of 1.9 for previous Omicron BA.1 infection. For a previous infection with a SARS-CoV-2 Alpha or Delta variant, our results were also similar to the Danish study’s protection estimates of 61.2% (95% CI: 49.1–70.4) and 73.8% (95% CI: 67.8–78.6) for, respectively, BA.5 and BA.2 after previous Alpha infection, a ratio of 1.5. For previous Delta infections, the Danish study observed protection estimates of 73.4% (95% CI: 65.7–79.3) and 84.2% (95% CI: 80.7–87.1) for BA.5 and BA.2, a ratio of 1.7, while we found an OR of 1.3 [14]. A pre-print from Portugal reported a similar difference between BA.4/5 and BA.2 regarding protection after a previous infection without vaccination (OR: 1.8; 95% CI: 1.3–2.5) or with primary vaccination (OR: 1.7; 95% CI: 1.4–2.0) both relative to unvaccinated without a previous infection [15]. Their estimate for booster vaccination with a previous infection (OR: 1.2; 95% CI: 1.0–1.5), however, was somewhat lower than our estimate. Similar to our results, vaccination status irrespective of previous infection status did not differ between BA.5 or BA.4/5 and BA.2 cases in the United Kingdom and Portugal [15,16].

Overall, we observed a reduction in protection conferred by previous infection for BA.4/5 relative to BA.2 and no changes in protection by vaccination. The reduction in protection from previous infection seems mainly driven by escape from BA.1-induced protection. Others have argued that the large number of reinfections with BA.5 after BA.1 is more the consequence of the larger numbers of BA.1 infections compared with the numbers of infections by earlier SARS-CoV-2 variants [17]. However, our data suggest that also immune escape plays a role in the large number of reinfections with BA.5. Still, the difference in escape from previous infection between BA.2 and BA.4/5 is smaller than the differences found between BA.1 and Delta [4], suggesting more escape between VOC than within the Omicron lineage.

A case-only analysis indicates whether protection against a variant changes relative to the reference variant, in our case BA.2 infection [18]. An advantage of our case-only approach on comparing levels of protection between variants is that it prevents bias from poor control selection. Previous studies used a similar design and found relative differences between earlier VOC [4,19,20]. However, our study has some limitations. Firstly, a considerable but unknown part of the previous infections will not have been registered because of lack of testing or symptoms, and this will have misclassified some individuals as not previously infected. This is likely to have diluted the actual differences in protection between previously infected and not infected individuals. Secondly, there is the possibility that the same infection is detected two times by PCR with a 30-day interval and we could therefore have misclassified some infections as reinfections. However, increasing the reinfection interval would also imply that we miss more reinfections. Thirdly, self-reported vaccination status could have led to some misclassification, although this is not likely to differ between variants. Fourthly, we could not distinguish BA.4 from BA.5 using TaqPath PCR data, although the majority of the SGTF cases were BA.5 infections (83.6%) and the spike sequences of BA.4 and BA.5 are identical and differences in anti-spike antibody escape are therefore not expected [9]. However, our results might differ slightly from estimates specific for BA.5 as 16.4% of the variants found among SGTF are BA.4 and other variants. Limiting our analysis to WGS data only would give us insufficient power due to small sample size. In addition, previous and current variants classified with TaqPath could sometimes be misclassified. However, as (non)-SGTF is almost always a very good predictor, the number of misclassifications is expected to be very small.

Despite the above differences between variants, protection conferred by previous infection with an Omicron variant in general is good, especially in combination with vaccination [3,14,17]. Vaccine effectiveness and protection by previous infection against severe disease is higher than against infection, also for the Omicron BA.2, BA.4 and BA.5 variants [22,23]. 

## Conclusion

Our results suggest a stronger reduction in protection against infection from previous infection against BA.4/5 compared with the BA.2 variant. This immune evasion is also observed within the Omicron lineage, especially for the first Omicron lineage that became dominant, BA.1. In contrast, no association between vaccination status and variant of infection was observed, indicating a similar vaccine effectiveness against infection with BA.2 and BA.4/5. Immune evasion within the Omicron lineage allows for repeat Omicron infections to occur and is therefore informative for considerations on vaccine updates and stresses the importance of studies on immune evasion by current and novel variants.

## Supplementary Data

1370000 Supplement
