# Supplementary material for: Higher risk of SARS-CoV-2 Omicron BA.4/5 infection than of BA.2 infection after previous BA.1 infection, the Netherlands, 2 May to 24 July 2022
Source: Euro Surveill. 2023 Feb 16;28(7):2200724. doi: 10.2807/1560-7917.ES.2023.28.7.2200724 (PMC9936591; doi:10.2807/1560-7917.ES.2023.28.7.2200724)
Supplement: Supplement [file 22-00724_EGGINK_Supplement.pdf]

Supplementary figures and tables for ‘Higher risk of SARS-CoV-2 Omicron BA.4/5 infection than of BA.2 infection after previous BA.1 infection, the Netherlands, 2 May to 24 July 2022 ’

This supplementary material is hosted by *Eurosurveillance* as supporting information alongside the article ‘Higher risk of SARS-CoV-2 Omicron BA.4/5 infection than of BA.2 infection after previous BA.1 infection, the Netherlands, 2 May to 24 July 2022 ’, on behalf of the authors, who remain responsible for the accuracy and appropriateness of the content. The same standards for ethics, copyright, attributions and permissions as for the article apply. Supplements are not edited by *Eurosurveillance* and the journal is not responsible for the maintenance of any links or email addresses provided therein.

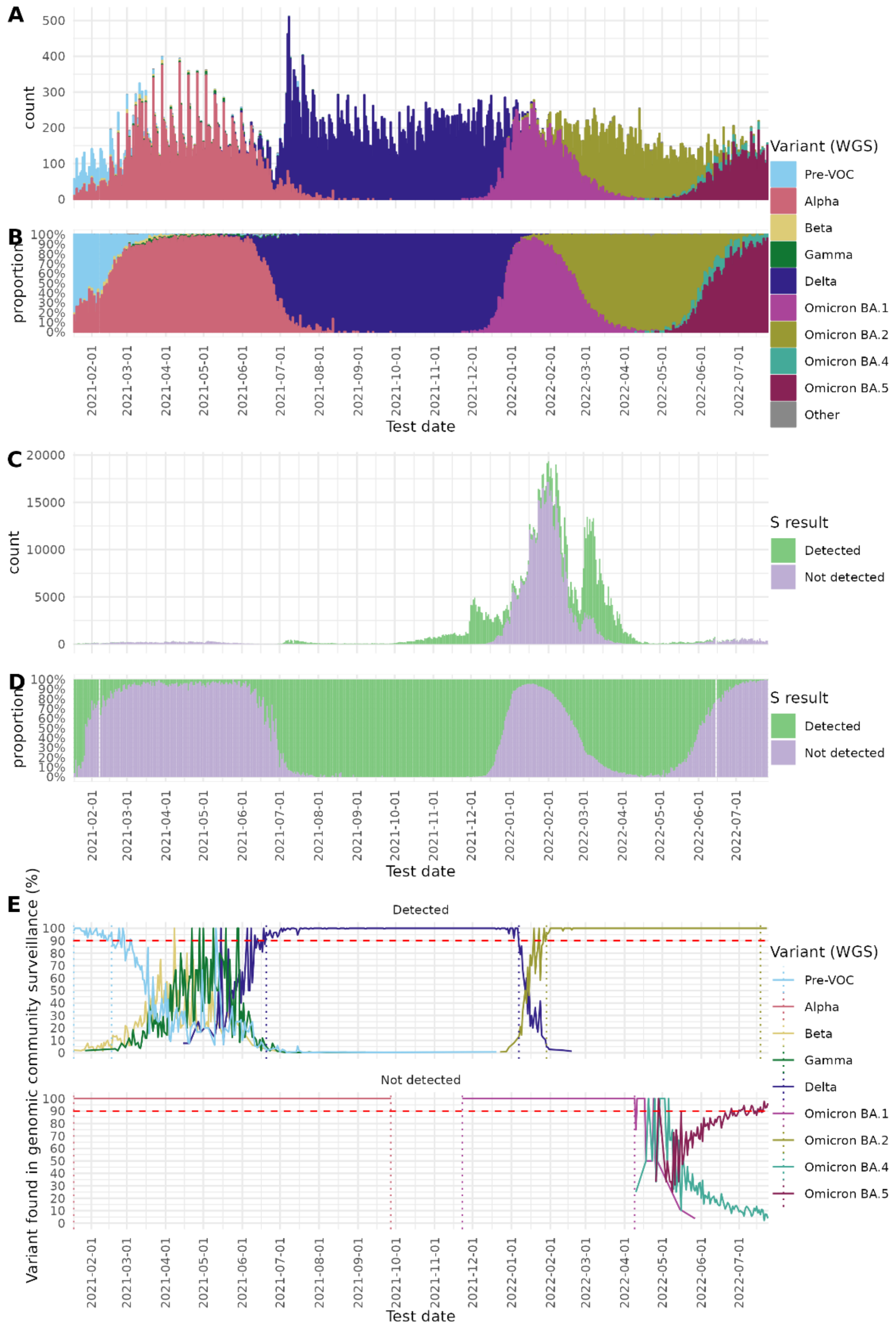

Fig S1 detection method of variant using SGTF. (A) number and (B) proportion of WGS variants found per date of testing. (C) number and (D) proportion of SGTF result detected per date of testing. (E) Proportion of the whole genome sequencing (WGS) typed variants found in national genome surveillance of community surveillance by SGTF status of the variant. Horizontal red striped line indicates the 90% threshold. Vertical dotted lines indicate the time periods used for previous variant detection using SGTF.

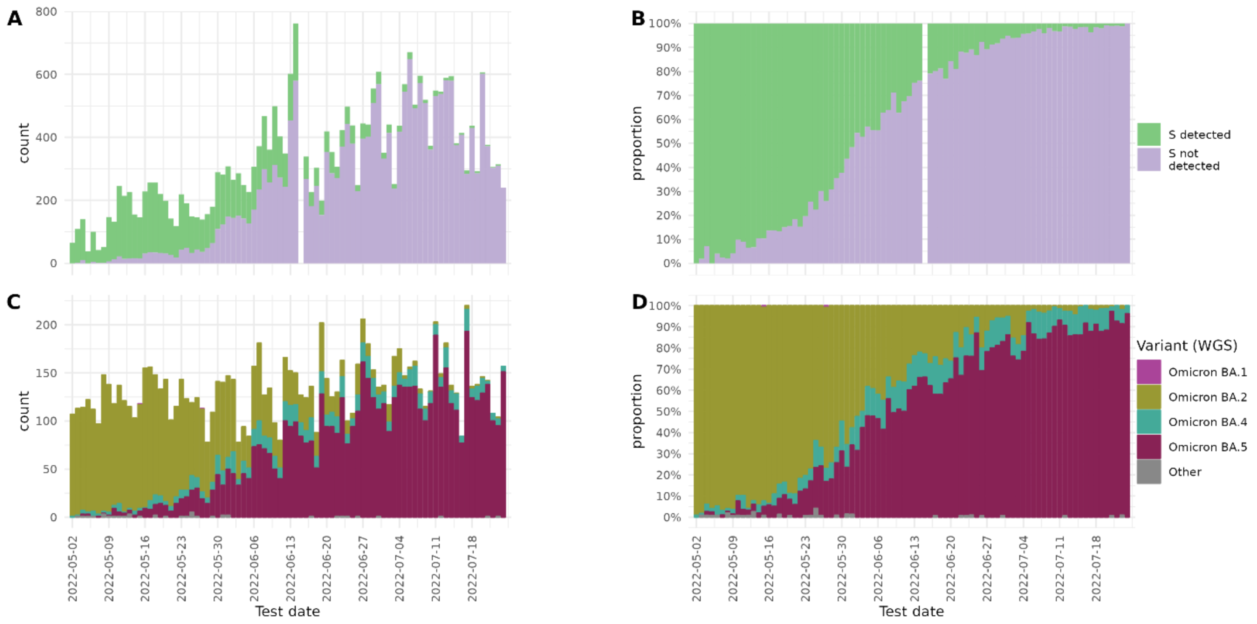

Fig S2 SGTF status and WGS typed variant found in community surveillance, the Netherlands, 2 May - 24 July 2022. Number (A) and proportion (B) of S-gene target failure (SGTF) and non-SGTF positive tests over time (n = 26888). Number (C) and proportion (D) of whole genome sequencing (WGS) typed variant found in national genome surveillance of community testing samples in the Netherlands (n = 11249).

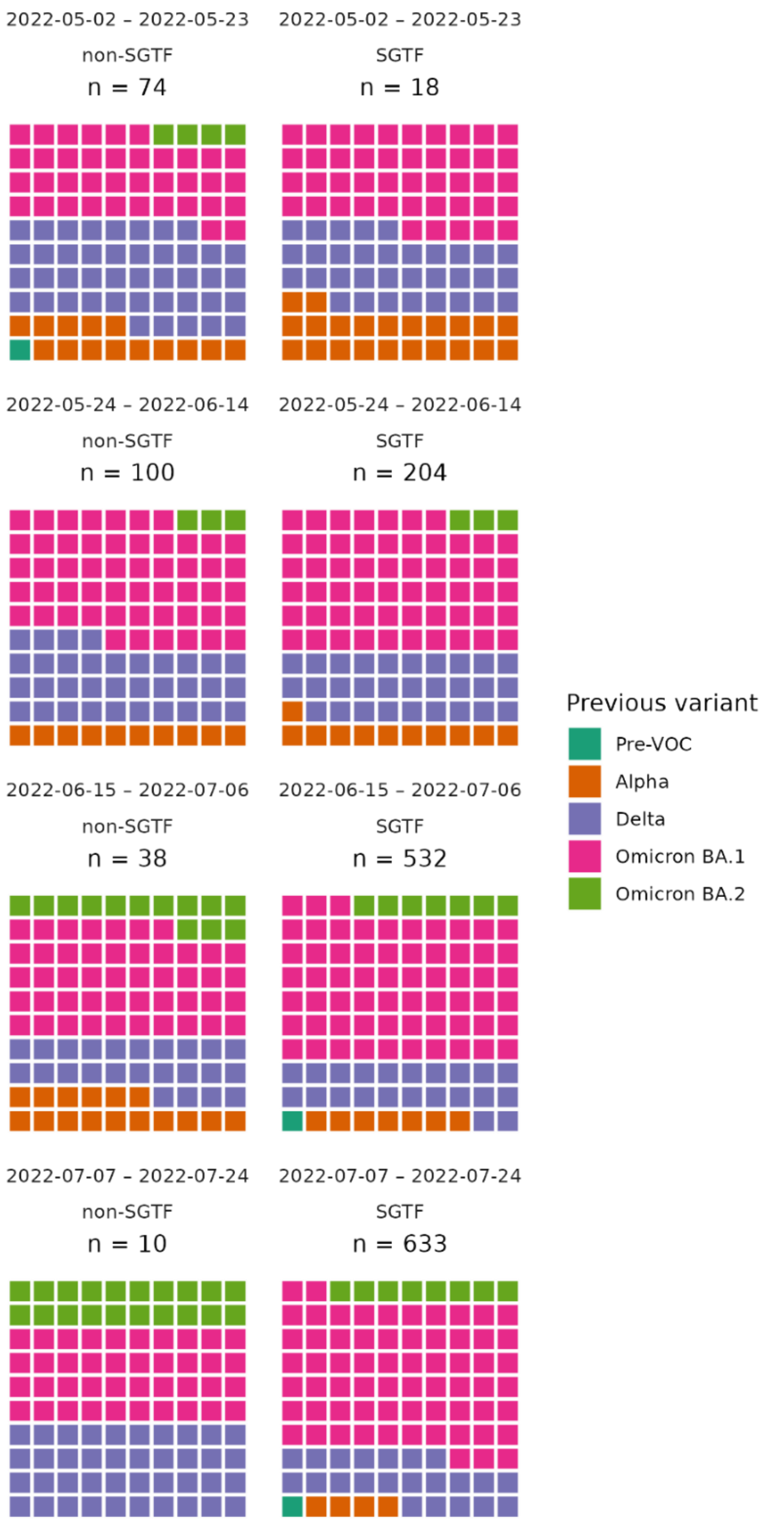

Fig S3 *Proportion of previous variants among reinfection pairs with a known previous variant for SGTF (BA4.5) and non-SGTF (BA.2) cases stratified by testing date (3 week groups).*

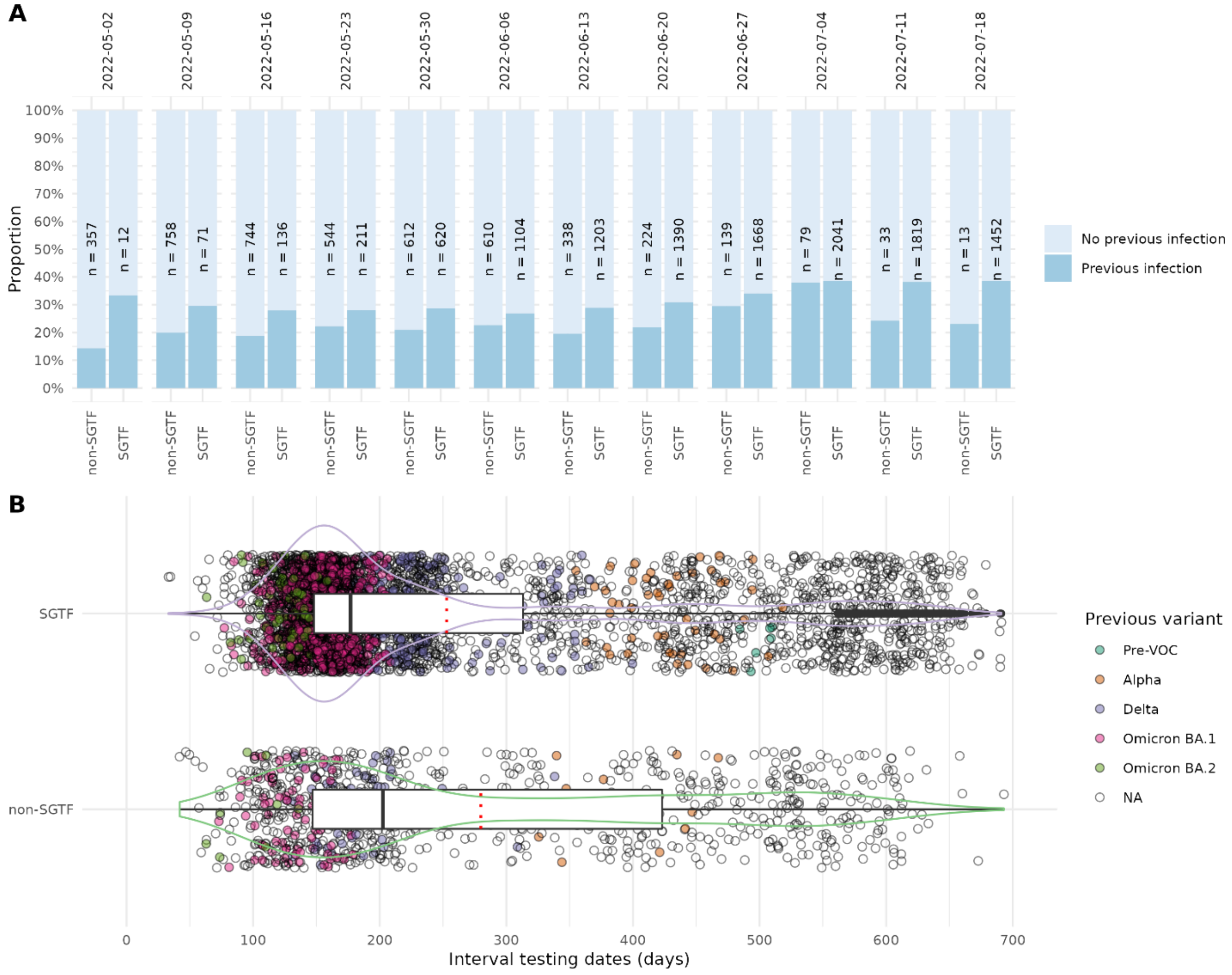

Fig S4 Complete case analysis on vaccination status. (A) Proportion of previous infections among non-SGTF (BA.2) and SGTF (BA.4/5) cases with known vaccination status, n= 16,178, the Netherlands, per week, 2 May - 24 July 2022. (B) Interval of testing dates in reinfections between previous and current infections of non-SGTF (with BA.2) and SGTF (BA.4/5) cases in days (n = 4,907). Current infections are colored to indicate the variant of the previous infection. Median is displayed in black (line) and mean in red (dotted).

	BA.2	%	BA.4/5	%	Adjusted OR (95%-CI)
No previous infection	3526	79,2	7745	66	Reference
Previous infection	925	20,8	3982	34	1.4 (1.3-1.6)
No previous infection	3526	95,9	7745	89,4	Reference
Pre-VOC	0	0	6	0,1	NA
Alpha	14	0,4	47	0,5	2.3 (1.1-5.1)
Delta	54	1,5	227	2,6	1.6 (1.1-2.4)
Omicron BA.1	75	2	580	6,7	1.7 (1.2-2.3)
Omicron BA.2	8	0,2	58	0,7	0.7 (0.3-2.0)

Table S1 Complete case analysis on vaccination status for the association between previous infection (n= 16,178, top panel), or previous variant (n =12,340, bottom panel), and BA.4/5 versus BA.2 infection, with no registered previous infection as reference group. Analyses are in individuals aged 18 years and older, adjusted for week of testing, sex and age group (18-29, 30-49, 50-69, and 70+ years).
